# Different Methylation Patterns of *RUNX2, OSX, DLX5* and *BSP* in Osteoblastic Differentiation of Mesenchymal Stem Cells

**DOI:** 10.22074/cellj.2015.513

**Published:** 2015-04-08

**Authors:** Majid Farshdousti Hagh, Mehrdad Noruzinia, Yousef Mortazavi, Masood Soleimani, Saeed Kaviani, Saeed Abroun, Ali Dehghani Fard, Maryam Mahmoodinia

**Affiliations:** 1 Department of Hematology, Tarbiat Modares University, Tehran, Iran; 2 Hematology and Oncology Research Center, Tabriz University of Medical Sciences, Tabriz, Iran; 3 Department of Medical Genetics, Tarbiat Modares University, Tehran, Iran; 4 Sarem Cell Research Center (SCRC), Sarem Women’s Hospital, Tehran, Iran; 5 Department of Hematology, Zanjan University of Medical Science, Zanjan, Iran

**Keywords:** DNA Methylation, Osteoblasts, Mesenchymal

## Abstract

**Objective:**

Runt-related transcription factor 2 (*RUNX2*) and osterix (*OSX*) as two specific osteoblast transcription factors and distal-less homeobox 5 (*DLX5*) as a non-specific one are of paramount importance in regulating osteoblast related genes including osteocalcin, bone sialoprotein (*BSP*), osteopontin and collagen type Iα1. The present study sets out to investigate whether epigenetic regulation of these genes is important in osteoblastic differentiation of mesenchymal stem cells (MSCs).

**Materials and Methods:**

In this experimental study, MSCs were differentiated to osteoblasts under the influence of the osteogenic differentiation medium. DNA and RNA were extracted at days 0, 7, 14 and 21 from MSCs differentiating to osteoblasts. Promoter regions of *RUNX2, OSX, DLX5* and *BSP* were analyzed by methylation-specific PCR (MSP). Gene expression was analyzed during osteoblastic differentiation by quantitative real-time polymerase chain reaction (PCR).

**Results:**

MSP analysis revealed that promoter methylation status did not change in *RUNX2, DLX5* and *BSP* during MSC osteoblastic differentiation. In contrast, *OSX* promoter showed a dynamic change in methylation pattern. Moreover, *RUNX2, OSX, DLX5* and *BSP* promoter regions showed three different methylation patterns during MSC differentiation. Gene expression analyses confirmed these results.

**Conclusion:**

The results show that in differentiation of MSCs to osteoblasts, epigenetic regulation of *OSX* may play a leading role.

## Introduction

Bone marrow contains both mesenchymal and hematopoietic stem cells. Mesenchymal stem cells (MSCs) have the potential of self renewal and can differentiate into several cell lineages including osteoblasts, chondrocytes, adipocytes and myoblasts (1). The use of MSCs in medicine has created much interest due to their multipotency and relative ease of accessibility. Their use has great potential in regenerative medicine and in tissue engineering (2). MSCs have demonstrated efficacy in multiple types of cellular therapeutic strategies, including treatment strategies in patients with osteogenesis imperfecta, hematopoietic recovery cardiovascular repair, lung fibrosis, spinal cord injury and bone tissue regeneration (3). Although MSCs are of ever-growing use in cell-based strategies, the mechanisms governing MSC self-renewal and multilineage differentiation have not been well understood and require further investigation. Therefore, in order to use MSCs in controlled and standardized ways, more knowledge about the molecular mechanisms underlying MSC commitment and differentiation is required.

Differentiation of MSCs in osteoblastogenesis is regulated by different kinds of morphogens, hormones, growth factors, cytokines and extracellular matrix (ECM) proteins. These external signals initiate several signaling cascades and transcription factors that mediate and control osteoblastogenesis. Several transcription factors are known to control bone development and osteoblast differentiation (3, 4).

Some transcription factors are specific to osteoblast differentiation and bone formation. Runt-related transcription factor 2 (*RUNX2*) and osterix (*OSX*) are two well-known osteoblast-specific transcription factors. However, several other non-osteoblast specific transcription factors have been identified to control osteoblast differentiation, including twist homolog 1 (*TWIST1*), zinc finger and BTB domain containing 16 (*ZBTB16*), distal-less homeobox 5 (*DLX5*) and MSH homeobox homolog 2 (*MSX2*) (4).

*RUNX2* [also known as core binding factor α 1 (CBFA1)] is the first described osteoblast-specific transcription factor and is key to osteoblast differentiation. *RUNX2* knockout mice show osteoblast differentiation arrest leading to the lack of osteoblast and bone development, but not cartilage (5, 6). In humans, heterozygous mutations in *RUNX2* cause cleidocranial dysplasia (CCD), a disorder characterized by hypoplasia or aplasia of the clavicles, short stature, supernumerary teeth, patent fontanelles and other changes in skeletal pattern and growth (7). Heterozygous *RUNX2* knockout mice show abnormalities mimicking CCD in humans (5).

*OSX* as a zinc finger-containing transcription factor expressed in osteoblasts plays a leading role in the osteoblast differentiation pathway (8). *OSX* belongs to the subgroup (Sp) of the Kruppel-like family of transcription factors, characterized by a three zinc-finger DNA-binding domain located close to the carboxy-terminus of the protein (9). It is proposed that *RUNX2* acts upstream of *OSX*, because studies have shown that in *RUNX2* null mice, *OSX* is not expressed whereas in *OSX* null mice, *RUNX2* is expressed (8).

*DLX5* is expressed in the early stages of bone synthesis and has been suggested that it plays a central role in osteogenesis regulation (10). A Recent study has shown that *DLX5* may have an important role in chondrogenesis and osteogenesis by controlling expression of some specific genes (11). Most of the early and late markers of osteoblast differentiation could be a direct target of *DLX5*.

*BSP* is one of the main components of mineralized tissues such as bone, teeth and calcified cartilage (12). Regulation of the *BSP* gene is important to bone matrix mineralization and tumor growth in bone (13, 14). Hence, as a potential nucleator of hydroxyapatite and as a specific marker of osteoblast and cementoblast differentiation, *BSP* has received considerable interest. However, due to its role in cell attachment and cell signaling, *BSP* is considered as a protein with multiple roles and importance in several pathologies.

Gene expression changes are crucial for the progression of stem cell differentiation. On the other hand epigenetic changes are important in the heritability and control of cellular gene expression pattern during differentiation. The dynamic nature of epigenetic changes has been shown in a recent study on different stem cells (15). Aranda et al. (16) showed that differentiation potential in stem cells is associated with epigenetic status changes. Our research group showed that ROR2 hypomethylates during osteoblastic differentiation of mesenchymal stem cells (17). Also, endothelial cell-specific genes [cluster of differentiation 31 (CD31)], CD144 are hypomethylated during adipose stem cells differentiation (18). Ezura et al. (19) showed that methylation status of chondrocyte signature genes (*SOX9, CHM1, FGFR3*) during chondrogenesis of MSCs are hypomethylated. Furthermore, a recent study has shown that human methylome changes dynamically during differentiation, and promoter hypomethylation correlates with transcription in all cell types (20).

In this research, we therefore evaluated the role of epigenetic regulation of *OSX, DLX5, BSP* and *RUNX2* in osteoblastic differentiation of MSCs. Moreover, the relation between methylation status and expression levels of these genes was explored during osteoblast differentiation.

## Materials and Methods

### Preparation, culture and osteogenic differentiation induction of human MSCs

In this experimental study, bone marrow-derived human MSCs were obtained from Stem Cells Technology Research Center (Tehran, Iran). MSCs were certified for positive differentiation potential for adipogenic, chondrogenic and osteogenic lineages by Stem Cells Technology Research Center. MSCs were analyzed by flow cytometry to confirm their identity as described before (21). Cells were re-suspended in Dulbecco’s Modified Eagle’s medium (DMEM)-low glucose (Gibco, Grand Island, NY, USA) supplemented by 15% (v/v) fetal bovine serum (FBS), 2-mM glutamine, 100 μg/ml of Streptomycin, 100 U/ml of Penicillin and plated in T75 polystyrene plastic cell culture flasks at the density of approximately 3×10^4^ cells/ml (22). When the adherent spindle-shaped fibroblastic cells reached 50-60% confluency, cells were harvested with 0.25% (w/v) trypsinethylenediaminetetraacetic acid (EDTA) solution and were plated in T75 cell culture flasks at a density of 3×10^4^ cells/cm.

Osteogenic differentiation medium was prepared by supplementing the growth medium with 5 mM β-glycerol phosphate, 50 μg/ml ascorbate-2-phosphate and 50 mg/ml dexamethasone (Sigma, St Louis, MO, USA). Cells were cultured and differentiated in both T75 culture flasks and 6-well plates. Growth and differentiation media were replaced twice a week. Undifferentiated MSCs and osteoblastic differentiated cells were harvested in days 7, 14 and 21 with trypsin-EDTA solution. Six-well plates were used for Alizarin Red Staining (ARS).

### Alizarin Red Staining (ARS)

For ARS, cells were washed twice with phosphate buffered saline (PBS) and fixed by formalin at room temperature for 10 minutes. Formalin was removed and the wells were washed twice with PBS and once with distilled water. ARS solution was then added and incubated at room temperature for 30 minutes. Finally, the wells were washed with distilled water until the background staining on the negative wells (wells containing MSCs) was fully cleared. Cells were examined by an inverted optical microscope (Nanjing Jiangnan Novel Optics Co., China).

### DNA extraction

DNA was extracted from both undifferentiated MSCs and osteoblastic differentiated cells were examined by DNA Extraction Kit (Roche, Germany) according to manufacturer’s instructions with minor modifications to increase DNA yield from calcified cells. Briefly, the cells were harvested by trypsin-EDTA solution and washed twice with PBS. Approximately, 3-4×10^6^ cells were harvested from each T75 flask. Then, the cell pellet was re-suspended in 200 μl PBS and transferred into a 1.5 ml microtube. By adding 200 μl binding buffer and 40 μl proteinase K, the solution was mixed immediately and incubated at 70˚C for 10 minutes. After incubation, 100 μl isopropanol was added and mixed well. A filter tube was inserted into a collection tube and samples were transferred into the filter tube. Then centrifugation for 1 minute at 8000×g, the filter tube was removed from the collection tube and combined with another collection tube. After that, 500 μl of inhibitor buffer was added to the filter tube and centrifuged for 1 minute at 8000×g. The washing procedure was repeated twice. Finally, the filter tube was inserted into a sterile 1.5 ml microtube and 50 μl of pre-warmed elution buffer was added into the filter tube followed by incubation for 10 minutes at room temperature, and centrifugation for 1 minute at 8000×g. Quality of extracted DNA was analyzed by agarose gel electrophoresis (Akhtarian, Iran). DNA yield and purity were evaluated by the absorbance of the elute at 260 nm and the ratio of absorbance at 260 nm and 280 nm was calculated.

### Methylation of genomic DNA with SssI methylase

A sample of peripheral blood-extracted DNA was methylated *in vitro* using Sss1 methylase (New England Biolabs, England) according to manufacturer’s instructions. Briefly, the reaction mixture consisted of 10 μl of 10x buffer (pH=7.9 at 25˚C), 0.5 μl of 32 mM S-adenosyl methionine (Sigma Chem. Co., St. Louis, MO, USA), 6 μl DNA, 2 μl of Sss1 methylase (4000 U/ml) and sufficient amount of distilled water for the mixture to reach 100 μl. The methylation mixture was incubated at 37˚C and DNA was extracted immediately using DNA Extraction Kit (Roche, Mannheim, Germany). Methylated DNA was used as a positive control for methylation-specific PCR (MSP).

### Candidate gene selection and primer design

Four candidate genes, as possible targets for epigenetic regulation through DNA methylation, were selected from 3 categories of genes: i. genes encoding specific osteoblast transcription factors, such as *RUNX2* and *OSX*, ii. a gene encoding non-osteoblast-specific but important transcription factor such as *DLX5* and iii. an osteoblast related gene such as *BSP*. Table 1 shows the characteristics of selected genes and CpG islands. A CpG island was defined as a DNA segment with a CpG content of 50%, longer than 200 base pair (bp) nucleotides, and an observation/expectation (Obs/Exp) CpG ratio over 0.6 (23). The MSP primers of the four genes were designed by MethPrimer software (24), and are shown in table 2. Because the two strands of DNA are no longer complementary after bisulfite modification, strand-specific primers are used for polymerase chain reaction (PCR) amplification. Usually, the sense strand is chosen for primer design. The following criteria were used in MSP primer design: A. for methylation mapping, it is important to focus on CpG island regions, thus, we chose M and U primers in CpG island regions. B. Selected primers had at least one CpG site within their sequence, and the CpG site preferably was located at the very 3΄-end of their sequence to maximally discriminate between methylated DNA and unmethylated DNA. C. Primers had a minimum number of non-CpG "C" in their sequence to amplify only the bisulfite modified DNA. Thus, primers were preferred with more non-CpG 'C's. D. The primer pair for the methylated DNA (M pair) and the pair for the unmethylated DNA (U pair) had the same CpG sites within their sequence.

**Table 1:** Characteristics of RUNX2, OSX, DLX5 and BSP and selected CpG islands


Gene symbol	Gene name	location	Sequenceaccession IDs	CpG No. in UCSC	CpG islandsize (bp)

**RUNX2**	Runt-related transcription factor 2	6p21	NM_00434	165	2226
**OSX**	Osterix	12q13.13	NM_001173467	116	1146
**DLX5**	Distal-less homeo box 5	7q21.3	NM_005221	109	1330
**BSP**	Integrin-binding sialoprotein	4q21.1	NM_004967	127	1179


UCSC; University of California Santa Cruz.

**Table 2:** Primer sequences used in MSP and real-time PCR analyses


Gene name	Primer name	Primer sequence	Band size (bp)

**RUNX2**	RUNX2MF	TTTCGGAAATTGTATACGGCGC	93
	RUNX2MR	AACAACGAATCTCGAACCTACG	
	RUNX2UF	GGTTTTGGAAATTGTATATGGTGT	96
	RUNX2UR	AAACAACAAATCTCAAACCTACA	
	RUNX2(RT)F	CCCCACGACAACCGCACCAT	289
	RUNX2(RT)R	CGCTCCGGCCCACAAATCTC	
**OSX**	OSXMF	GTATCGGATAGGCGGAGATC	128
	OSXMR	AAACTAATCTAAACGAAACGACGAC	
	OSXUF	GGTATTGGATAGGTGGAGATTG	130
	OSXUR	AAAACTAATCTAAACAAAACAACAAC	
	OSX(RT)F	GCCAGAAGCTGTGAAACCTC	161
	OSX(RT)R	GCTGCAAGCTCTCCATAACC	
**DLX5**	DLX5MF	TTGGGGAATAAAGGTATACGTTATC	150
	DLX5MR	ACATAACTTCTTAACGATAAACCGC	
	DLX5UF	GGGGAATAAAGGTATATGTTATTGG	147
	DLX5UR	CATAACTTCTTAACAATAAACCACA	
	DLX5(RT)F	ACCAACCAGCCAGAGAAAGA	259
	DLX5(RT)R	TCTCCCCGTTTTTCATGATC	
**BSP**	BSPMF	CGATTCGTAGCGGTAGATGTAC	107
	BSPMR	ACGAAACGCAAAAAAAATACG	
	BSPUF	GTGATTTGTAGTGGTAGATGTATGA	110
	BSPUR	AAACAAAACACAAAAAAAATACAAA	
	BSP(RT)F	TGCCTTGAGCCTGCTTCCT	79
	BSP(RT)R	CTGAGCAAAATTAAAGCAGTCTTCA	
**GAPDH**	GAPDH(RT)F	CGTCTTCACCACCATGGAGA	300
	GAPDH(RT)R	CGGCCATCACGCCACAGTTT	


MSP; Methylation-specific PCR, PCR; Polymerase chain reaction, RUNX2; Runt-related transcription factor 2, OSX; Osterix, DLX5; Distal-less homeo box 5, BSP; Bone sialoprotein and GAPDH; Glyceraldehyde-3-phosphate dehydrogenase.

### RNA isolation and cDNA synthesis

Total RNA was extracted from both undifferentiated MSCs and osteoblastic differentiated cells using an RNeasy plus Mini kit (Qiagen, USA) according to manufacturer’s instructions. Briefly, harvested cells were disrupted and homogenized. The homogenized lysate was transferred to a gDNA Eliminator spin column and was centrifuged for genomic DNA elimination. After adding ethanol, total RNA was bound to an RNeasy spin column. Then washing, total RNA was eluted by RNase-free water. The integrity of RNA was determined by gel electrophoresis prior to reverse transcription.

Three μl of extracted RNA were converted to cDNA using 20 pmol random hexamer primer, 4 μl 5X reaction buffer [250 mM Tris-HCl, 250 mM KCl, 20 mM MgCl_2_, 50 mM Dithiothreitol (DTT)], deoxyribonucleotide triphosphate (dNTP) Mix (10 mM), M-MuLV Reverse Transcriptase (Fermentas, Lithuania) in total volume of 25 μl. Reverse transcription was carried out for 60 minutes at 42˚C. Reverse transcriptase was inactivated by heating at 70˚C for 10 minutes.

### Quantitative real-time PCR analysis

Quantification of mRNA expression for candidate genes was performed by quantitative real-time PCR using the LineGene 9600 florescent quantitative detection system (Hangzhou Bioer Technology, China). Real-time PCR reactions were performed by Quantifast SYBR Green PCR kit (Qiagen, USA) according to the manufacturer’s instructions. Briefly, real-time PCR reactions were performed in a total volume of 25 μl containing 12.5 μl of 2X QuantiFast SYBR Green PCR Master Mix, 1 μl of cDNA from individual samples, 0.5 μl of each primer (see Table 2) and 10.5 μl RNase-Free Water. Real-time PCR reactions were carried out in triplicate. The real-time PCR thermocyclic conditions included an initial step of 5 minutes at 95˚C, followed by 40 cycles of 95˚C for 10 seconds and 60˚C for 1 minute. Gene expression levels were normalized to *glyceraldehyde-3-phosphate dehydrogenase (GAPDH)* expression (ΔCt=C_tgene of interest_-Ct_GAPDH_). Results were reported as relative gene expression (2^-ΔCt^).

### Sodium bisulfite treatment and methylation-specific PCR

For bisulfite treatment, 1 μg of DNA in a total volume of 50 μl H_2_O was denatured by NaOH (final concentration of 0.2 M) for 10 minutes at 37 ˚C. Thirty μl of freshly prepared 10 mM hydroquinone (Merck) and 520 μl of freshly prepared 3.5 M sodium bisulfite (pH=5, Merck) were added to the samples. Each sample was incubated under mineral oil at 50˚C for 16 hours. Modified DNA was purified with Qiagen DNA purification columns according to the manufacturer’s instruction and eluted into 200 μl of elution buffer. Desulfonation was achieved by NaOH (final concentration of 0.3 M) treatment for 5 minutes at room temperature. DNA was precipitated by ethanol and was dissolved in 30 μl distilled water and used immediately for MSP or stored at -20˚C.

MSP was performed using specific primers capable of distinguishing between methylated and unmethylated DNA sequences. The PCR mixture contained 10x PCR buffer (500 mM KCl; 100 mM Tris-HCl (pH=8.3); 15 mM MgCl_2_), dNTP mix (each at 1.25 mM), primers (0.5 μM of each primer), 2 mM MgCl_2_, 1-4% dimethyl sulfoxide (DMSO), 1.25 unit of Taq DNA Polymerase (Fermentas, Lithuania) and bisulfite-modified DNA (50 ng) or untreated genomic DNA (gDNA; 50-100 ng) in a final volume of 25 μl. The reproducibility of MSP was tested through repeating the reactions by HotStarTaq Plus Master Mix Kit (Qiagen, USA). Briefly, 12.5 μl HotStarTaq Plus Master Mix (HotStarTaq Plus DNA Polymerase, PCR Buffer, 1.5 mM MgCl_2_ and 200 μM each dNTP), primers (0.5 μM of each primer), 2.5 μl 10x CoralLoad PCR buffer, bisulfite-modified DNA (50 ng) or unmodified DNA (50-100 ng) in a final volume of 25 μl was set up for PCR reactions. When using Ferementas Taq DNA polymerase, reactions were manually hot-started at 95˚C for 5 minutes before the addition of 1.25 unit of Taq DNA polymerase. Amplification was carried out in a MyCycler thermal cycler (Bio-Rad) for 35 cycles (30 seconds at 95˚C, 30 seconds at the annealing temperature from 52 to 62˚C, and 30 seconds at 72˚C), followed by a final extension at 72˚C for 10 minutes.

Unmodified DNA and methylated DNA were used as negative and positive controls respectively. Ten μl of each PCR was directly loaded onto a 1.5% agarose gel and a non-denaturing 8% polyacrylamide gel, stained with ethidium bromide, and directly visualized under ultraviolet (UV) illumination.

### Statistical analysis

To normalize the results obtained from genes expression, GAPDH gene was used as an internal control. The relative quantitation values have been obtained based on cycle threshold (CT) method and were calculated using the formula 2^-ΔΔCt^. Statistical analysis was also performed using software statistical package for social science (SPSS)-15 and t test. In addition, the results were obtained from three different repetition samples [(mean ± standard deviation (SD)]. The p<0.05 was considered statistically significant.

## Results

### Osteoblast differentiation confirmation

**Fig.1:**
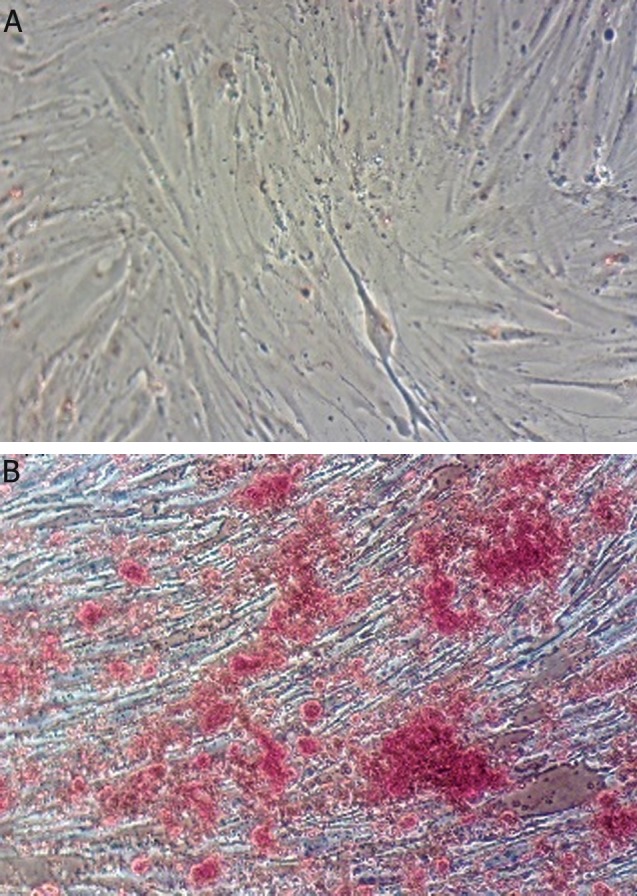
ARS for calcium deposition detection. A. Undifferentiated MSCs are negative for ARS and no deposition of calcium and B.Osteoblastic cells at day 21 of differentiation that are positive for ARS and calcium deposits are represented as red granules. ARS: Alizarin red staining and MSCs; Mesenchymal stem cells.

ARS of osteoblastic differentiated cells confirmed the presence of calcium deposition characteristic of osteogenic cells whereas undifferentiated MSCs were negative for ARS (Fig.1).

### Quantitative expression of RUNX2, OSX, DLX5 and BSP during osteoblastic differentiation

**Fig.2:**
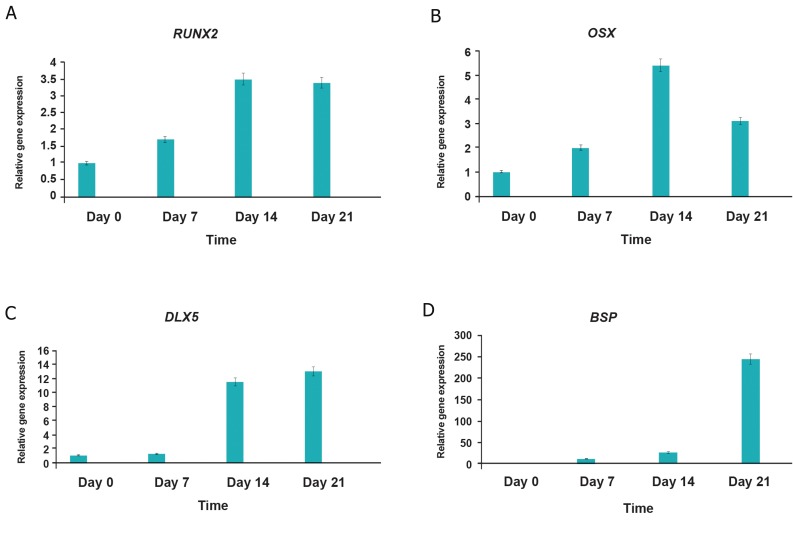
Real-time RT-PCR for relative gene expression of (A) RUNX2, (B) OSX, (C) DLX5 and (D) BSP during osteoblastic differentiation of MSCs. Day 0; Undifferentiated MSCs, Days 7, 14 and 21; Osteoblastic differentiated cells days 7, 14, 21, respectively. Gene expression of RUNX2 and OSX increases slightly whereas gene expression of DLX5 and especially BSP increases markedly during osteoblastic differentiation of MSCs. Triplicates were made for each approach. RT-PCR; Reverse transcription polymerase chain reaction, MSCs; Mesenchymal stem cells, RUNX2; Runt-related transcription factor 2, OSX; Osterix, DLX; Distal-less homeobox 5 and BSP; Bone sialoprotein.

Gene expression analysis showed that the expression of *RUNX2, OSX, DLX5* and *BSP* was upregulated during osteoblastic differentiation of MSCs. Gene expression of *RUNX2* in the first, second and third week of osteogenic differentiation, compared with the undifferentiated MSCs, showed 1.7-fold, 3.5-fold and 3.4-fold increase in expression respectively (Fig.2A). *OSX* expression during osteogenic differentiation, compared with the undifferentiated MSCs, showed 2-fold, 5.4-fold and 3.1-fold increase in expression in weeks 1, 2 and 3 of differentiation respectively (Fig.2B). *DLX5* was over-expressed 1.2-fold, 11.5-fold and 13-fold (Fig.2C) and *BSP* expression showed 11.7-fold, 26.9-fold and 244-fold increase in expression in the same intervals respectively (Fig.2D).

### Methylation status of CpG islands in the promoter regions of RUNX2, OSX, DLX5 and BSP in osteoblastic differentiation of MSCs

**Fig.3:**
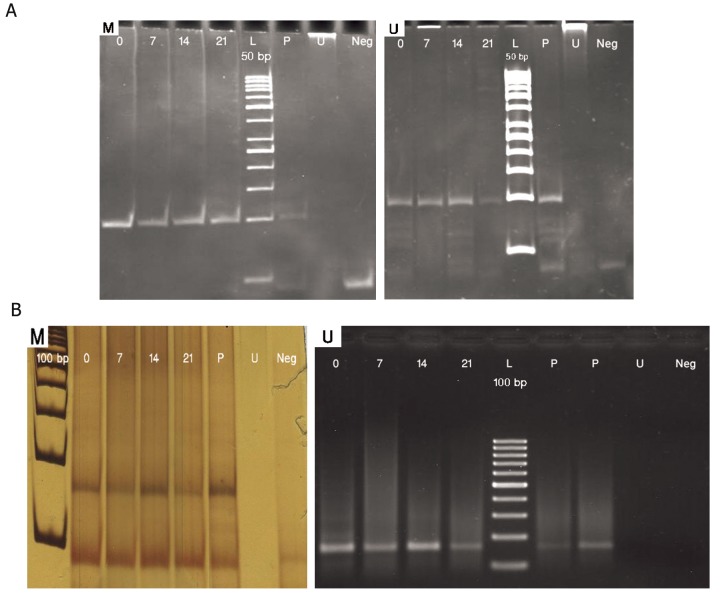
MSP results with methylated (M) and unmethylated (U) primers for (A) RUNX2 and (B) DLX5. Day 0; Undifferentiated MSCs, Days 7, 14 and 21; Osteoblastic differentiated cells days 7, 14, 21, respectively, P; Positive control (methylated and SBS treated DNA in M experiments and only SBS treated DNA in U experiments), U; SBS untreated DNA as negative control for MSP and Neg; No DNA as negative control. Amplicon size in RUNX2: mexperiment (93 bp) and U experiment (96 bp). Amplicon size in DLX5: M experiment (150 bp) and U experiment (147 bp). MSP; Methylation-specific PCR, RUNX2; Runt-related transcription factor 2, DLX; Distal-less homeobox 5, MSCs; Mesenchymal stem cells, SBS; Sodium bisulfite.

**Fig.4:**
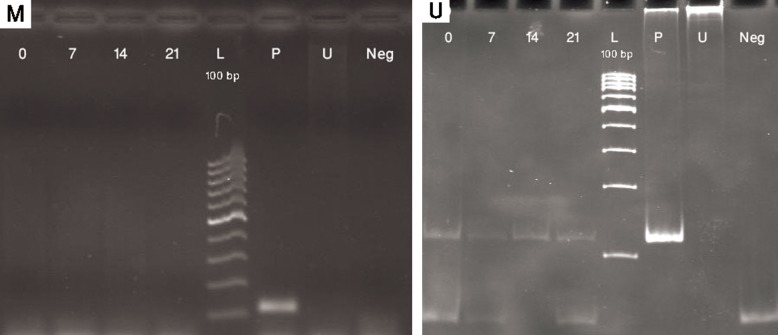
MSP results with methylated (M) and unmethylated (U) primers for BSP. Day 0; Undifferentiated MSCs, Days 7, 14 and 21; Osteoblastic differentiated cells days 7, 14, 21, respectively, P; Positive control (methylated and SBS treated DNA in M experiments and only SBS treated DNA in U experiments), U; SBS untreated DNA as negative control for MSP and Neg; No DNA as negative control. Amplicon size in BSP: M experiment (107 bp) and U experiment (110 bp). MSP; Methylation-specific PCR, BSP; Bone sialoprotein, MSCs; Mesenchymal stem cells and SBS; Sodium bisulfite.

The methylation-specific PCR analysis did not show any significant change in methylation pattern within the analyzed regions of *RUNX2, DLX5* and *BSP* promoters in neither the undifferentiated state nor the osteoblastic differentiated state of MSCs. *RUNX2* and *DLX5* promoter analysis showed that these genes were partially unmethylated both in MSCs and differentiated osteoblasts (Fig.3). *BSP* promoter, on the other hand, was totally unmethylated in both mesenchymal stem cells and during differentiation (Fig.4). Thus, methylation status of *RUNX2, DLX5* and *BSP* promoter regions did not change during osteoblastic differentiation.

**Fig.5:**
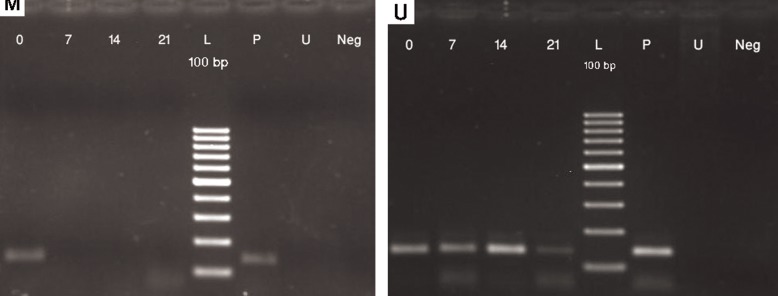
MSP results with methylated (M) and unmethylated (U) primers for OSX. Day 0; Undifferentiated MSCs, Days 7, 14 and 21; Osteoblastic differentiated cells days 7, 14, 21, respectively, P; Positive control (methylated and SBS treated DNA in M experiments and only SBS treated DNA in U experiments), U; SBS untreated DNA as negative control for MSP and Neg; No DNA as negative control. Amplicon size in OSX: M experiment (128 bp) and U experiment (130 bp). MSP; Methylation-specific PCR, OSX; Osterix, MSCs; Mesenchymal stem cells and SBS; Sodium bisulfite.

**Fig.6:**
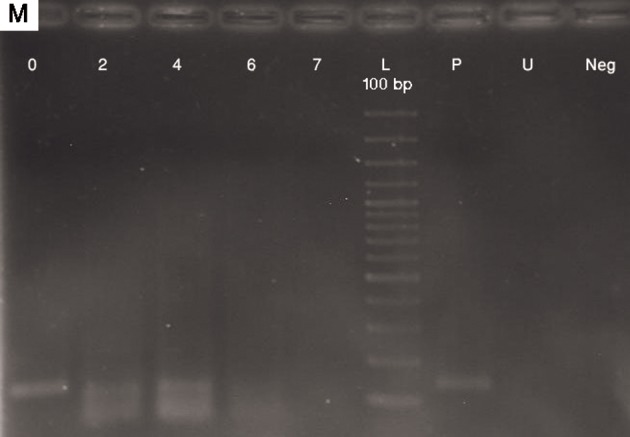
MSP results with methylated (M) primers for OSX in the first week. Day 0; Undifferentiated MSCs, Days 7, 14 and 21; Osteoblastic differentiated cells days 7, 14, 21, respectively, P; Positive control (methylated and SBS treated DNA), U; SBS untreated DNA as negative control for MSP and Neg; No DNA as negative control. Amplicon size in OSX: M experiment (128 bp). MSP; Methylation-specific PCR, OSX; Osterix, MSCs; Mesenchymal stem cells and SBS; Sodium bisulfite.

In contrast, *OSX* showed a change in methylation pattern while MSCs were gradually differentiated to osteoblasts. In day 0 of differentiation, MSCs were totally methylated till day 4 of differentiation. On day 4, differentiating MSCs were partially unmethylated and by the 7^th^ of differentiation, cells were totally unmethylated. *OSX* promoter remained unmethylated afterwards (Figs.5, 6).

## Discussion

In this study, we characterized different methylation patterns in the differentiation process of MSCs. The epigenetic mechanisms alter chromatin (DNA and associated proteins) in ways that change the availability of transcription factors to genes required for their expression. These alterations are related to DNA methyltransferases, which add a methyl group to cytosine in the dinucleotide CpG, and a wide variety of chromatin modifying elements. Osteogenic differentiation of MSCs is accompanied by various epigenetic modifications at chromatin and DNA levels (25,26). It has been shown in another study that certain genes in stem cell are gradually methylated during osteoblastic differentiation (27). On the contrary, the osteogenic specific genes are upregulated with chromatin activating factors (25). This effect has also been reproduced at the DNA level. Yeo et al. (28) showed that the promoter regions of *OCT4* and *NANOG*, but not *SOX2, REX1* and *FOXD3*, undergo significant methylation during human embryonic stem cells (hESCs) differentiation thus substantially repressing stem cell marker genes. 

In this study we found, in concordance with previous works, that osteogenic specific genes *RUNX2* and *OSX* are upregulated in osteogenic process. Liu et al. (29) showed that after 2 weeks of osteogenic differentiation, the osteogenic transcription factors *OSX* and *RUNX2* showed more than 2-fold and 5-fold increase in expression respectively. Our findings show that *RUNX2* and *OSX* expression are upregulated more than 3-fold and 10-fold respectively. However, this gene expression upregulation was not accompanied by DNA demethylation as it would have been expected. This result might be a reflection of the technique used in this research. MSP is a non-quantitative technique and cannot measure the proportion of methylated to unmethylated DNA when an incomplete methylation is present (30). Ezura et al. (19) showed that the methylation status of promoter CpG islands is preserved in critical genes, such as *SOX9* and *RUNX2*, during chondrogenesis. This finding is in agreement with our work which showed an absence of change of methylation pattern of *RUNX2* during differentiation. However, Kang et al. (31) showed that *RUNX2, BGLAP,* and *CDKN2A* in the bone marrow *MSC*, and *PPARγ2* in the adipose tissue stem cells were strongly expressed and hypomethylated in the transitional CpGs. 

The present work confirmed previous findings showing that in lineage restriction during development of MSCs, DNA methylation is as important as transcription factors and chromatin modifications. These factors are essential in differentiation of stem cells, which in turn requires withdrawal from the cell cycle and re-establishment of a program of gene expression (25). We showed that the promoter region of *OSX* becomes gradually hypomethylated during osteoblastic differentiation. This hypomethylation correlates well with gene over-expression where *OSX* is upregulated more than 10-folds during osteoblastic differentiation of MSCs. Mukherjee and Rotwein showed that expression of *OSX* in C3H10T1/2 (mouse embryonic fibroblasts) cultured in osteogenic medium with or without BMP2 began in days 7 and 5 of differentiation respectively (32). Also, Valenti et al. (33) showed that *OSX* expression in differentiated osteoblasts of peripheral blood MSCs was detectable after 3 days of differentiation. We showed in this study that *OSX* is upregulated as early as the 7^th^day of differentiation of MSCs. 

DNA demethylation is considered to be accompanied by gene expression changes (17). However, DNA methylation change in this study was not found to be accompanied by gene expression change for all of the studied genes. *OSX* for example is totally methylated at the end of the first week, while the peak of expression upregulation is at the end of the second week. This may imply that mechanisms other than DNA methylation might accompany gene expression change. It has been further confirmed that chromatin level epigenetic changes are important in stem cell commitment to differentiated cells (25). *BSP* on the other hand is considered as a late marker of osteoblastic differentiation of MSC. We found a late onset upregulation of *BSP*, confirming a late role for this protein, but the methylation study showed no change in methylation status of the promoter of this gene. This again implies that other mechanisms of gene expression regulation are present in *BSP* transcription. 

## Conclusion

This work provides the first assessment of CpG methylation in promoter regions of osteoblasticrelated genes (i.e. *RUNX2, OSX, DLX5* and *BSP*) in relation to osteoblastic differentiation potential in human MSCs. We confirm the previously suggested role for *RUNX2, OSX, DLX5* and *BSP* in osteoblastic differentiation of MSCs. We further show that methylation change is not the main epigenetic mechanism in *BSP* upregulation. However *OSX, RUNX2* and *DLX5* expression are influenced by DNA methylation. Quantitative techniques can further elaborate on the exact role played by methylation in transcription regulation of these genes and such further studies are thus warranted. 
